# Quality of life after stroke in Pakistan

**DOI:** 10.1186/s12883-016-0774-1

**Published:** 2016-12-03

**Authors:** Wardah Khalid, Shafquat Rozi, Tazeen Saeed Ali, Iqbal Azam, Michael T. Mullen, Saleem Illyas, Qamar un-Nisa, Nabila Soomro, Ayeesha Kamran Kamal

**Affiliations:** 1 Fogarty Cerebrovascular Research Fellow, The International Cerebrovascular Translational Clinical Research Training Program (Fogarty International Center, National Institutes of Health) and Aga Khan University, Karachi, Pakistan; 2 Department of Community Health Sciences, Epidemiology & Biostatistics, Director Clinical Research Track (CRT), Aga Khan University, Karachi, Pakistan; 3 School of Nursing and Midwifery (SONAM), Karachi, Pakistan; 4 Department of Community Health Sciences, Aga Khan University Hospital, Karachi, Pakistan; 5 Department of Neurology, Perelman School of Medicine at the University of Pennsylvania, Philadelphia, USA; 6 Development Centre DOW University of Health Sciences, Karachi, Pakistan; 7 Department of Neurology, DOW University of Health Sciences, Karachi, Pakistan; 8 Department of Medicine, Lecturer, Section of Neurology, Aga Khan University, Karachi, Pakistan; 9 Institute of Physical Medicine and Rehabilitation, DOW University of Health Sciences, Karachi, Pakistan; 10 The International Cerebrovascular Translational Clinical Research Training Program, Fogarty International Center and the National Institute of Neurologic Disorders and Stroke, Aga Khan University, Stadium Road, 74800 Karachi, Pakistan; 11 Department of Medicine, Aga Khan University, Karachi, Pakistan

**Keywords:** Stroke Specific Quality of life scale (SSQOLS), Post-stroke depression, LMIC (Lower middle income country), FGD`S (Focus group discussion) Sequential Mix Methods, Triangulation, Epidemiology, Complications, Chronic Disease

## Abstract

**Background:**

There is very little information about the quality of life (QOL) of stroke survivors in LMIC countries with underdeveloped non communicable health infrastructures, who bear two thirds of the global stroke burden.

**Methodology:**

We used a sequential mix methods approach. First, a quantitative analytical cross-sectional study was conducted on 700 participants, who constituted 350 stroke survivor and their caregiver dyads. QOL of stroke survivor was assessed via Stroke Specific Quality of Life Scale (SSQOLS) whereas QOL of caregivers was assessed through RAND-36. In addition; we assessed complications, psychosocial and functional disability of stroke survivors. Following this quantitative survey, caregivers were qualitatively interviewed to uncover contextually relevant themes that would evade quantitative surveys. Multiple linear regression technique was applied to report adjusted β-coefficients with 95% C.I.

**Results:**

The QOL study was conducted from January 2014 till June 2014, in two large private and public centers*.* At each center, 175 dyads were interviewed to ensure representativeness. Median age of stroke survivors was 59(17) years, 68% were male, 60% reported depression and 70% suffered post-stroke complications. The mean SSQOLS score was 164.18 ± 32.30. In the final model severe functional disability [adjβ -33.77(-52.44, -15.22)], depression [adjβ-23.74(-30.61,-16.82)], hospital admissions [adjβ-5.51(-9.23,-1.92)] and severe neurologic pain [adjβ -12.41(-20.10,-4.77)] negatively impacted QOL of stroke survivors (*P < 0.01*).

For caregivers, mean age was 39.18 ± 13.44 years, 51% were female and 34% reported high stress levels. Complementary qualitative study revealed that primary caregivers were depressed, frustrated, isolated and also disappointed by health services.

**Conclusion:**

The QOL of Stroke survivors as reported by SSQOLS score was better than compared to those reported from other LMIC settings. However, Qualitative triangulation revealed that younger caregivers felt isolated, depressed, overwhelmed and were providing care at great personal cost. There is a need to develop cost effective holistic home support interventions to improve lives of the survivor dyad as a unit.

**Trial registration:**

NCT02351778 (Registered as Observational Study).

**Electronic supplementary material:**

The online version of this article (doi:10.1186/s12883-016-0774-1) contains supplementary material, which is available to authorized users.

## Background

In the last decade, the overall incidence of stroke increased by 20% in low to middle income countries (LMIC) [1–3]. Pakistan is an LMIC country where about one out of four adults have either Hypertension, Type II Diabetes or Cardiovascular disease and these highly prevalent risks make them uniquely stroke prone [4]. Although robust large scale epidemiologic data is lacking, a reported prevalence of 4.8% translates into 4 million people living with stroke in Pakistan [5]. No large scale epidemiological studies are available to determine the true incidence of stroke in Pakistan. Estimated annual incidence is 250/100,000, translating to 350,000 new cases every year [6]. Not all strokes are assessed by a neurologist. General physician and internists also take care of patients with strokes.

The social, physical and psychological consequences of stroke are devastating [7–10]. These consequences are described by studies that elaborate Quality of Life(QOL). QOL is defined as “Individual’s perceptions of their position in life in the context of the culture and value systems in which they live and in relation to their goals, expectations, standards and concern.” However, studies about QOL from LMIC regions are lacking [11].

In Pakistan, two thirds of the population pays out of pocket for health care expenses [12]. There are no inpatient rehabilitation services or organized chronic home support services, and the stroke caregiver is often a close family member [13]. We aim to describe the QOL of stroke survivors and their caregivers in Pakistan, where the realities and context of care differ from other health care systems. To the best of our knowledge, this is the first study in Pakistan to report QOL after stroke. This study served as a hypothesis generating study, as we did not have any previous data from our population, based on which we would had suggested any specific hypothesis to test regarding QOL. For the analytical purposes of this first study we simply hypothesized that there would be an association between Quality of life of Stroke Survivors and independent predictors: Socio-demographic, Psychological, individual Stroke Characteristics and Caregivers Factors. These hypotheses were generated on existing stroke conceptual models [14].

Since QOL is a multidimensional concept, therefore, we used a mixed method approach with a quantitative survey followed by Focus Group Discussion (FGD`S) and In-Depth Interviews thus using a qualitative open ended approach to triangulate and complement our quantitative observations [15].

## Methods

Sequential mix methods approach was adopted to describe the complex dimensions of QOL [16]. Firstly, an analytical cross sectional study was conducted to determine QOL and its important associations then followed by qualitative FGD`S and In-Depth Interviews (IDI) to further characterize and describe locally relevant LMIC themes.

### Quantitative study methods

#### Setting

The QOL of Stroke Survivors Study was conducted at two tertiary hospitals, one private (Aga Khan University Hospital, AKUH) and one public (DOW University hospital, DUHS), based in Karachi Pakistan [17, 18]. Karachi is a metropolitan microcosm of Pakistan with a population of estimated 23.5 million inhabitants and a mix of all ethnicities [19]. These two hospital systems were chosen as together they serve all socio-economic and ethnic strata living in Pakistan encompassing those who can afford fee for service and those who are indigent. Pakistan has a mixed health system comprised of both public and private sector fee for services systems.

Public sectors provide treatment free of cost or at very subsidized rates [15]. There are a few private hospitals in Karachi that manage stroke patients. It is almost impossible for the majority of the population to afford these private services as around 60.3% lives on under $2 a day with a single earning member in the whole family [4, 6].

### Recruitment

All stroke survivors with their care givers visiting neurology out-patient clinics of aforementioned hospitals were approached. The interviews were performed at their respective Centers from Feb 2014 till May 2014.

### Eligibility criteria

Stroke survivors and their primary caregivers were interviewed as a dyad. Stroke survivors greater than 18 years of age with confirmed diagnosis of stroke (confirmed by a neurologist with radiological evidence by either CT scan and/or MRI) with the following eligibility criteria were recruited.

Inclusion Criteria:

- ▪ Stroke of more than 1 month.
- ▪ Stable non fluctuating stroke with no acute ongoing neurologic developments.
- ▪ With an identified primary care giver greater than 18 years of age.
- ▪ No preexisting disability prior to stroke (defined by mRS =0).

Exclusion Criteria:

- ▪ Post procedural stroke like CABG, angiography and post-operative stroke.
- ▪ Global aphasia and/or unable to communicate.
- ▪ Dementia on Minimental State Examination (of <22), (Due to inability to directly communicate)
- ▪ History of ongoing psychoactive substance abuse, presence of psychiatric morbidity before and after stroke which specifically includes manic disorders, schizophrenia
- ▪ Associated terminal illnesses like renal failure or end stage cancer.

### Assessment tools used in the QOL study

The questionnaire was formulated based on WHO conceptual framework to be comprehensive in our approach to QOL [14]. (Additional file 1: Figure S1). Age, gender, educational status, occupation, marital status, family status was reported as demographic data. For socioeconomic indicators, World Bank Wealth index questionnaire was adopted [20]. Stroke survivors Medical complications, Post-stroke complications were noted at the time of interview [21] and stroke sub-types were evaluated through medical reports [22]. Neurologic severity was assessed by National Institute of Health Stroke Scale (NIHSS) [23]. Barthel Index, Modified Rankin Score, and questions of Functional Assessment Measures (FAM) assessed functional disability [23]. Dementia was ascertained by Mini-mental State Examination and depression was ascertained by Beck Depression Inventory [24, 25].

The presence of social support was evaluated by the Enriched Social Support Inventory [26]. The Stroke Specific Quality Of Life Scale (SSQOL) [27] was used to measure domains of energy, family roles, language, mobility, mood, personality, self-care, social roles, thinking, vision, upper-extremity function and work productivity. For primary caregivers, RAND-36 was used to evaluate Health related QOL. It assess eight health concepts which include physical functioning, role limitations caused by physical health problems, role limitations caused by emotional problems, social functioning, emotional well-being, energy/fatigue, pain, and general health perceptions [28]. Primary care givers stress level was assessed by the Perceived Stress Scale [29].

### Tool validation and reliability

After due permissions from authors of tools, we performed content validation with a committee of international and national experts in stroke, rehabilitation, social sciences, epidemiology, biostatistics and psychiatry. Content Validity refers to how accurately an assessment tool taps into the various aspects of the specific construct in question. Content Validity Index (CVI) was calculated for each tool. CVI quantifies the level of content validity by calculating the percentage agreement between the experts. CVI is calculated by dividing the proportion of items judged by the expert panel to have content validity by the total number of items in the scale [30]. In our study CVI for relevance ranged from 0.9 to 1 and for clarity ranged from 0.68 to 0.99 for different scales. In literature CVI of greater than 0.8 indicates high level of agreement among the experts [31]. Based on expert advice during the process of content validation, questions were verbalized and explained in a more contextual way to enhance their clarity for better understanding. The questionnaire was translated and back translated to Urdu and then used for data collection. Post analysis reliability of scales was determined by Cronbach`s-alpha that ranged from 0.66 to 0.94. Higher scores depict more reliability of the scales and it is recommended to be 0.70 or greater [32] but lower scores are also reported to be used in literature [33]. [The detail results of content validation will be published separately]. However these scale statistics indicate that the adapted, content validated tools were reliable, relevant and clear.

### Sample size calculation

The sample size was calculated by one population mean formula , based on mean QOL scores with standard deviation of ±15.2 [34], 5% level of significance with precision of 2 and for adjusting for 10% non-response rate, the minimum sample size was estimated to be 245 dyads. We thus recruited 350 dyads. The allocated sample size for two study site was calculated based on the total number of patients visiting these sites per month, a total of 175 dyads were required from each study site to have a proportionate representation of participants. Purposive sampling technique was employed at both sites.

### Statistical analysis plan

Statistical analysis was performed on STATA VERSION 12. To report baseline and descriptive statistics, for normally distributed continuous variables, Mean ± S.D were reported and for non-normally distributed variables, Median with Inter-quartile range (IQR) was reported. For categorical variables, frequencies with percentages were reported. For socioeconomic status, Factor Component Analysis was performed on proxy variables. Simple linear regression analysis was performed and variables based on the *p*-value of <0.25 and were selected for multivariable analysis. Any factor that was clinically relevant was considered of biologic importance. The independent variables at univariate level were selected based on a *P*-value of <0.25. This *P*-value cutoff is mentioned by Hosmer and Lemeshow [35]. The reason behind this selection was to identify more variables at univariate level for the main effect model. To assess the independent effect of associated factors on QOL multiple linear regression analysis was performed and adjusted β-coefficients with their 95% CI were computed. All plausible interactions were also assessed for inclusion in multivariable model. Model adequacy was assessed on final multivariable model by plots of residuals against fitted values and normal probability plots.

### Qualitative study methods

To complement the findings of the quantitative study, the primary caregiver experience was further elaborated with sequential qualitative interviews and discussion [16]. We chose to interview the primary givers separately as their responsibilities are unique in our setting and we wanted an open ended, qualitative method to elaborate their experiences with a sense of security and privacy.

### Participant criteria

The interviews were conducted, only with primary care givers with following criteria:

Age greater than 18 years, able to speak and understand Urdu, care givers of aphasic patients and demented patients were included as these patients constitute an important part of the larger stroke population and we wanted to know their perspective regarding QOL and we felt that they would be better suited to an open ended design.

### Qualitative study procedures

The study employed purposive sampling technique. Recruitment was performed from all clinics to have variability within the sample. Interviews were conducted in June 2014 till the thematic saturation level of the content and perception of caregivers was achieved and no other further information was added. In total ten interviews were conducted, seven In-depth and three FGD`S. To clarify, seven In-depth interviews with one participant each and three Focus group interviews with at least four participants were performed. In total qualitative assessment was made on 20 participants. The interviews were only voice recorded with permission of participant and informed consent was obtained. All interviews were first transcribed then recordings were compared with the transcripts for verification to increase the accuracy and were translated into English within 10 days. Strict privacy and confidentiality was maintained for all data and recordings.

### Interview guide

A thematic interview guide was formulated with inputs from all regional experts and international input. A Pre-interview session was performed with the caregivers to evaluate whether the context and content were clear to them. Their suggestions were incorporated into the thematic guide. Important open ended questions included were: What are the important factors that affect life of stroke patients? What changes came in your life as a primary care giver? How can we improve the QOL of stroke survivors?

### Qualitative analysis

Qualitative manual content analysis was performed to explain the manifest content and the latent content [36]. Content analysis is a step by step systematic process [37]. Initially the narrative data were read several times by the researchers which enabled them to comprehensively understand the text and comprehend the meaning of content according to local context. In the next step the narrative data was combined into meaningful and understandable units which were labeled as codes [37]. The coding process was performed by two researchers separately, these codes were then compared, discussed and consensus was reached within the research team. In the third step the codes were analyzed in detail and they were grouped together into meaningful sub-categories and were labeled with appropriate title. Further the sub-categories were merged into broader categories. In the last step categories were pooled into major themes. With the discussion of the research team codes were finalized, sub-categories and categories were identified and themes emerged. To achieve trustworthiness, obtaining data on sensitive perceived factors demanded through planning and understanding. The researchers were experts in the local traditions, meaning, communication and use of specific language, metaphors of word interpretation and understanding specific to the local context (greater than 10 years of direct experience with stroke care in the region). Credibility was achieved by selection of context, well-structured questions and participants. Transferability was gained by purposive sampling of participants with diverse characteristics like gender, age, socioeconomic status, different ethnic background and care givers of aphasic and demented patients; Dependability was achieved by conducting interviews within one month to make sure that the phenomena of the study remained unchanged [37]. Conformability and consensus was achieved through discussion on codes, sub-categories, categories and themes within the research team.

### Ethics and human subject protections

Study protocol was approved by both institutional review boards with Aga Khan University Hospital Ethics Review Committee with ERC Ref #:2762-Med-ERC-13 and Dow University of Health Sciences Institutional Review Board with reference number of IRB-416/DUHS/-13. Interviewers (physician and sociologists) were ethics certified and trained prior to any direct contact with participants. Stroke patients and caregivers provided individual written informed consent to participate in the study. In cases where the patient’s ability to consent was impaired, consent for these participants was obtained from next of kin in the presence of a witness. Strict confidentiality and privacy was ensured, and the dyads were interviewed separately, to respect individual confidentiality. The study was also registered online as an observational study at the clinical trails.gov website with ClinicalTrials.gov Identifier is NCT02351778.

### Results

#### Participant numbers and flow

In total, 700 individual participants were interviewed. There were 350 stroke and caregiver dyads. There were 175 dyads interviewed from each study site. 46 patients refused for participate in study and 75 patients were excluded from both study sites. (Additional file 2: Figure S2).

### Socio-demographics

The demographic and stroke characteristics are described in Table 1. The median(IQR) age of stroke survivors was 59(17) years. There were more male participants around 68.8%and 64%majority had good social support from families. The median (IQR) duration of stroke was 13.83 (25.11) in months.

**Table 1:** Baseline characteristics of stroke patients

Characteristics	N (%)
Age yrs^b^	59(17)
Male Sex	241 (68.8)
Education	
No School	113 (32.2)
Up till 10 grade	121 (34.5)
Above 10 grade	116 (33.1)
Socioeconomic Status^d^	
Low income group	183 (52.2)
Middle income group	51 (14.5)
High income group	116 (33.1)
Family status	
Joint family	224 (64)
Nuclear family	126 (36)
Marital status	
Married	281 (80.2)
Widowed &Widower	53 (15.1)
Unmarried	12 (3.4)
Divorced & Separated	4 (1.1)
Social Support by Enriched Social Support Inventory	
Low social support	126 (36)
Good social support	224 (64)
Health Care Access Factors	
Received Medical Coverage From Any Organization	41 (11.7)
Presence Of Health Care Professional Attendant	22 (6.2)
Received Rehabilitation Service	88 (25.1)
Stroke Sub-Types	
Atherosclerotic	269 (76.8)
Hemorrhagic	71 (20.2)
Unknown	10 (2.8)
Duration of Stroke in months^b^	13.83 (25.11)
Hypertension	278 (79.4)
Diabetes	120 (34.2)
Dyslipidemia	114 (32.5)
CVD(Angina, MI)	61 (17.4)
Smoking	91 (26)
Tobacco Usage	110 (31.4)
Central Obesity^c^	
Male	156 (44.5)
Female	83 (23.7)
Modified Barthel Index for Functional Dependency	
Minimal dependency	122 (34.8)
Mild dependency	76 (21.7)
Moderate dependency	70 (20)
Severe dependency	66 (18.8)
Total dependency	16 (4.5)
Functional Assessment Measure FAM^a^	65.7 ± 12.5
Modified Rankin Score for Disability	
Normal	3 (0.8)
No significant disability	74 (21.1)
Slight disability	81 (23.1)
Moderate disability	66 (18.8)
Moderately severe disability	105 (30)
Severe disability	21 (6)
Severity of Stroke assessed by NIHSS^a^	3(5)
Depression	
Normal	137 (39.1)
Mild mood disturbances	135 (38.5)
Borderline depression	58 (16.5)
Moderate depression	19 (5.4)
Severe depression	1 (0.2)
Mild Dementia	52 (14.8)
Post-stroke complication	251 (71.7)
Pressure Ulcer	8 (2.2)
Hospital Admission	75 (21.4)
Neurologic pain	
Mild to moderate pain	101 (28.8)
Severe pain	14 (4)
Hemiplegic Shoulder	96 (27.4)
Fall Events after Stroke	136 (38.8)
Urinary tract infection	21 (6)
Dysphagia	48 (13.7)
DVT	4 (1.1)
Mean Quality of life Score ^a^	164.18 ± 32.30

^a^Mean ± S.D

^b^Median (Inter-quartile range)

^c^The Asian cutoff in males of >90cms and in females of >80cms of abdominal circumference is considered as central obesity

^d^ Factor component analysis was performed to assess Socioeconomic Status

### Post -stroke complications and mean QOL scores

#### Complications

Sixty point eigthy five percent of stroke survivors reported depression and 71.70%suffered at least one post-stroke complication. Median (IQR) NIHSS score of survivors was 3(5). The mean QOL score of stroke participants was 164.18 ± 32.30.

### Caregiver descriptors

Female’s caregivers were more than half 51.14% of the sample population. The mean age of caregivers was39.18 ± 13.44 and 51.40% had changed their working hours to take care of stroke survivors. Around 34.29% of caregivers reported stress, whereas Health Related Quality of Life (HRQOL) assessed by RAND-36 showed mean scores in the 70s in physical functioning, energy, physical health and emotional well-being which reflect a good score on QOL. (Table 2)

**Table 2:** Primary caregiver baseline characteristics

Characteristics	N (%)
Age in years^a^	39.18 ± 13.4
Female Sex	179 (51.1)
Relationship of primary care givers to stroke patients	
Children	147 (42)
Spouse	126 (36)
Sibling	37 (10.5)
Parents	6 (1.7)
Others^c^	34 (9.7)
Education	
Illiterate	51 (14.5)
Matriculate	117 (33.4)
Above 10 years	182 (52)
Employment status	
Employed	135 (38.5)
Housewife	135 (38.5)
Unemployed	11 (3.1)
Retired	18 (5.1)
Others^d^	51 (14.5)
Stress of primary care giver	
Likely high stresses	120 (34.2)
Mean Stress level of primary care givers^a^	16.67 ± 7.44
Health related quality of life of primary care givers assessed by RAND-36	
Physical functioning^b^	80 (35)
Role Limitation due to physical health^b^	75 (25)
Role Limitation due to emotional problems^b^	66.6 (66.6)
Energy^a^	71.5 ± 19.5
Emotional well-being^b^	72.2 (15.4)
Social functioning^a^	66.3 ± 26.2
Pain^a^	67.2 ± 21.2
General health^a^	67.4 ± 24.1
Left job because of providing care	20 (5.7)
Change in working hours because of direct providing care	180 (51.4)

^a^Mean ± S.D

^b^Median(Inter-quartile range)

^c^Others category for relationship consists of daughter in law, grandchild, nephew, niece, sister in law, brother in law, son in law

^d^ Others category for employment status include Businessman, landlord, farmer, student

### Factors affecting QOL

Simple linear regression showed stroke survivor age, gender, education, socioeconomic group, marital status, family status, health care services, hypertension, smoking status, functional disability, depression, dementia, post-stroke complications, severity of stroke were significantly associated with the QOL of stroke survivors (*P*-value of <0.25). For caregiver characteristics, their age, employment status, relationship to patient, change in working hours, stress and HRQOL were also significantly associated with QOLof stroke survivors (*P* value of <0.25).

In final multivariable model, independent variables which were significantly associated with QOL of stroke survivors included moderate to severe disability, depression, increased level of independence, severity of stroke, severity of neurologic pain, hospitals admission with dementia (Table 3). The final model was adjusted for site of study. QOL of stroke survivors decreases by -33.77 (95% C.I; -52.44, -15.22) with every one scale increase in functional disability assessed by Modified Rankin score. QOL also decreases by -23.74 (95% C.I; -30.61, -16.82) with increase in level of depression. For every one unit increase in FAM score QOL increases by 0 .98 (95% C.I; 0.74, 1.22). Increase in severity of stroke also decreased QOL by-1.81 (95% C.I;-2.37, -1.26) for stroke survivor Additional file 3.

**Table 3:** Multivariable analysis of factors associated with quality of life of stroke survivors

	Adjusted β-Coefficient (95%CI)	*P*-value
Functional Disability		
No significant disability	-9.67 (-25.48 to 6.04)	0.22
Slight disability	-17.74 (-33.61 to -1.83)	0.02
Moderate disability	-21.27 (-37.53 to -5.07)	0.01
Moderately-Severe	-30.27 (-47.30 to -13.27)	<0.01
Severe disability	-33.77 (-52.44 to -15.22)	<0.01
Functional independence level (FAM)	0.98 (0.74 to 1.22)	<0.01
Severity of stroke(NIHSS)	-1.81 (-2.37 to -1.26)	<0.01
Depression		
Mild mood disturbances	-10.15 (-13.51 to - 6.81)	<0.01
Borderline depression	-16.75 (-21.25 to -12.23)	<0.01
Moderate depression	-23.74 (-30.61 to -16.82)	<0.01
Severe depression	-26.88 (-54.71 to 1.36)	0.06
Hospital Admissions	-5.51 (-9.23 to - 1.92)	<0.01
Neurologic pain		
Mild to moderate pain	-0.60 (-3.83 to 2.66)	0.72
Severe pain	-12.41 (-20.10 to - 4.77)	<0.01
Mild Dementia	-4.17 (-8.54 to 0.13)	0.06

The final model is adjusted for site

Adjusted R-Square of the final model is 0.82

*P*-value of the overall model is <0.01

The final model explains 82% of the variability in the outcome variable (Adjusted r^2^ = 0.82). All plausible interactions were found to be insignificant. Model assumptions were checked and residuals were plotted for normality and homoscadiscity. The residuals plots were normally distributed and no heteroscadicity was found out.

### Qualitative results

There were three important themes that we identified and described by this method (Table 4). These narrations are directly from caregiver interviews. The baseline characteristics of FGD`s and In-depth interviews are presented in Additional file 4: Table S2 of online supplementary appendix.

**Table 4:** QOL themes emerging through focus group discussion (FGD`S) and in-depth interviews

Theme	*Category*	Sub-category
Impact of stroke on life of patient	*Impact on independent status of patient*	1. Change in life because of functional dependence.
	*Impact on psychosocial behavior of patient*	1. Patient’s depression.
		2. Patient’s alterations in behavior.
		3. Patient’s reaction regarding their life.
	*Suffering of patient after stroke*	1. Uncertainty about future, shock and dismay.
Effect on life of primary care givers	*Change in life of primary care giver*	1. Change in personality of primary care giver guilt, anger and self-blame.
		2. Increased responsibility.
	*Primary care giver distress*	1. Primary care giver worries and frustration.
		2. Effect on health of primary care giver.
		3. Reaction of family towards patient condition after stroke.
	*Primary care giver desire*	1. Wish support and burden sharing.
Quality of life	*Perception of quality of life*	1. QOL may depend on physical independence, isolation and loneliness.
		2. QOL is affection/ encouragement and giving hope to patients.
	*How to improve quality of life of stroke patients*	1. Psychosocial, spiritual teachings and workshops with group activities to give confidence to patient.
		2. Unfamiliarity with how to improve patient’s QOL.
		3. Encourage primary care givers to improve QOL.
		4. Proper education of the society to except and help disable people.
		5. Restoration of physical functioning is QOL.
	*Future demands*	1. Proper health management team to give holistic care to patient and family
		2. Need of GP system to manage most of the conditions of the patients.
	*Primary care giver QOL*	1. Improvement of primary care giver QOL.
		2. Encouragement and guidance for primary care giver.
		3.Establishing a tele-health service.

### Stroke survivors QOL in LMIC settings

The loss of independence is deeply felt and perhaps because of stigma of disability and paralysis, stroke survivors are even often reduced to tears over their state of dependency. The patients are worried about their recovery. They are concerned about their treatment expenses. They report hopelessness “*He is fed-up of his dependency and thinks death is better than this kind of life.”* Patients are frustrated and angry. The families think that they have been “*influenced by the occult or black magic”*. They cannot accept their disease in rational terms. Survivors feel very lonely. Survivors miss their previous life. One care giver responded: “*There are sudden restrictions and limitation in their lives which are physical, mental and social. All these sociological, psychological and financial issues increase the magnitude of damage which makes them feel as if they are imprisoned”*.

### Caregiver s life after stroke in LMIC setting

All caregivers want to support their loved ones, but require support themselves. A care giver wished *that if she had a sister she would have helped her in taking care of her mother.* Caregivers feel weak, tired, deprived of sleep because of the continuous care and physical work. Their personal health is neglected. Majority of young caregivers suffered from altered eating habits, developed Type II DM and Hypertension. They also reported denial and difficulty accepting disability, according to one care giver *“death is better for his father as compared to his miserable life”.* Caregivers report stress, tiredness, anger and depression. They felt uninformed when they performed every caretaker related skill like positioning and NG feeding. The families have developed conflicts among themselves. One son responded that *“he wants to go somewhere or end his life”* because he has additional responsibility of family which he cannot fulfill like marriage of girls, education of children. There is a shift in responsibility now on young caregivers who are often young college going children. They are home bound as there are no alternate alarm or reporting systems and their work commitments and studies are profoundly affected.

### Perceptions on what is QOL and how life may be better after stroke

“*QOL is love, caring and encouragement*.” Stroke patients should not be confined and need to enjoy and explore the world. QOL may encompass reintroducing activities survivors cannot perform like reading, listening and socialization. Speech is also an important part of QOL. There is a need to have a health system where there is easy access. Doctors and medical team should have skills of “*counseling and discuss in detail the treatment plan”.* Doctors should “*manage other co-morbid of the patients … There should be a counseling team*.” “*Changes in environment like closer parking areas to clinics, proper bathroom facilities …. can help stroke patients*.” Caregivers should be encouraged and mentally prepared for this task. Training courses and guidance on how to care, understand aphasic patient’s needs, how to manage patients on NG feed, positioning and other medical conditions are required. Participants demanded tele-health services. All primary care givers responded *“that our QOL will automatically improve if condition of our loved ones gets better”.* Psychosocial therapy was requested with spiritual and faith healing as complement to medical therapy to bring improvement in lives and target depression. According to one daughter “*My mother improved more while getting rehabilitated at rehab center rather than at home as it motivates her to perform better in presence of other stroke patients.”* Another caregiver reported“Educate *society on how to deal with disability or disabled people. Media should play a responsible role”. “There is a need to broaden our thinking, read and to take help from internet to adopt changes in our life and to respect disabled people”.* There was a need articulated to raise self-esteem of patients so that they can be a part of the society.

### Triangulation of data

Triangulation is a powerful technique that facilitates validation of data through cross verification from two or more sources or combination of several research methods in the study of the same phenomenon [38]. In our study to have a deeper insight regarding the phenomenon of QOL we validated the finding of our quantitative data and qualitative interviews through methodological triangulation [39]. Functional dependence, depression and stress appeared to be important mediators of QOL in triangulation. Qualitative analysis revealed the stress and isolation of caregivers. Additionally, feedback on how to improve QOL was much greater in the open ended sessions.

## Discussion

QOL is a significant healthcare outcome relevant to communities and healthcare systems. It is not easy to describe and requires a multidimensional approach, encompassing domains of physical, mental and psychosocial state [34]. Despite its clear importance in chronic life altering disorders like stroke with pervasive disability, it has not been systematically investigated or reported from LMIC settings, where stroke is a major public health problem, populations are vulnerable and health systems are disorganized and/or lack the resources necessary to respond to chronic disease [40, 41]. An understanding of context is necessary to interpret these observations. Pakistan is the sixth most populous country in the world (185 million people) [42]. Its demographic transition of raising non-communicable epidemic and increased life expectancy (65 years) has resulted in a double burden of communicable and non- communicable disorders (NCDs) like strokes [43]. According to Global Burden of Disease (GBD) 2010 statistics, NCDs accounted for 77% of age standardized deaths [4]. Annual projections estimate an increase of 125–144 yearly vascular deaths from 2010 to 2025 per 100,000, translating into actual numbers of 231,000–307,200 persons annually and a cumulative expected premature mortality burden of up to 3.87 million individuals [2, 4, 44]. The reported lifetime prevalence of stroke symptoms in Pakistan is about 19%, with an estimated 7 million persons affected [45]. Despite these staggering numbers, the total per person annual expenditure on health is less than 18$, and there have been negligible specific investments [12, 40]. Approximately 78% of population pay out of pocket for health care and there is not a single comprehensive inpatient rehabilitation unit in Pakistan and no demonstrable examples of health care provision solutions for chronic disabling disorders in a system plagued by acute care responsiveness acute care trauma, infections and maternal and child care needs [12]. We report an understanding of how stroke survivors and caregivers fare when they are confronted with the challenge of managing a complex condition with little institutionalized support. Our sampled stroke survivors (Table 1) are comparatively younger as compared to the West where the reported age of stroke is about a decade later [46–48]. This increased survival with DALYs in a resource strapped setting points to the huge societal impact of stroke [49]. We have found that stroke survivors were young, mostly depressed and their QOL was profoundly influenced by increased functional dependency, depression and neurologic pain [46–48]. More than two thirds of sample population lived in joint family systems with good social support. Our study mean SSQOL score of 164.18 ± 32.30 was better as compared to Brazil with mean score of 139.7 ± 38.4 [50] and south east Nigeria with mean score of 156.71 ± 41.64 [51]. Perhaps this system may have been explanatory for the relatively better SSQOL scores, at the expense of caregiver burden.

When we elucidated the primary caregiver experience, we were able to appreciate the personal cost of being the primary caregiver. We were able to compare caregivers on the Perceived Stress Scale. The mean stress level of caregivers assessed by Perceived Stress Scale in our sample is 16.67 ± 7.44 which is higher as compared to 13.2 ± 7.2 as reported in the West [47]. Majority of the care givers were young, more than half of them were female in contrast to the older male stroke survivor. Qualitative results show that our caregivers are frustrated, isolated, physical burdened and face severe financial crises. The caregivers want to be navigated by a responsive health system. Qualitative interviews also reported the stress and frustration of families as they were not able to afford basic health facilities for their families. Only a quarter had access to any rehabilitation service which points to the unavailability, expense and dearth of rehabilitation services.

To the best of our knowledge, this is the first detailed mix methods study conducted in Pakistan that details the experience of those who suffer stroke in an LMIC setting with poor infrastructure for chronic care support. We performed a detailed quantitative assessment on 700 participants with locally validated instruments whose reliability was established apriori. We spend at least 1.5 h on interviews and thus we report 1050+ hours of feedback and observation. We did not observe any participant fatigue as there was a great need articulated that these findings must be shared. We attempted to be inclusive to socio-demographic determinants by equally interviewing participants in centers where all strata and ethnicities may seek care. We performed 10 qualitative interviews and included the range of disability by including caregivers of severely disabled participants who could not communicate in our quantitative interviews phase. Since we were contextually relevant, we evaluated primary care givers as they are the ones that compensate for our resource strapped health systems. We performed triangulation with qualitative methodology to explain and validate our observations and to uncover themes regarding important domains which could not be covered by standardized tools.

There are several limitations to this study. Our cross-sectional design does not enable assessment of changes in QOL temporal to stroke occurrence. This study was conducted in institutionalized settings and the results are not generalizable to community settings for those patients who do not report to hospitals where QOL may be much worse than our current observations.

## Conclusion

Stroke affects lives profoundly in LMIC countries of both survivors and their primary caretakers. The stroke survivor is young, usually male, relatively functionally compromised, depressed, has little access to organized rehab and despite these challenges reports a moderate score. The primary caregiver is younger, usually female, bears a personal cost of abandoning either a life role of a mother or a job to take direct responsibility of care, and most accept their role despite reporting depression, stigma and social isolation. For those designing interventions, chronic care models need to incorporate both survivors and caregivers in their rubric to actually improve lives after stroke.

## Additional files

Additional file 1: Figure S1. Conceptual Framework (Adopted from the WHO [World Health Organization]). (DOCX 33 kb)
Additional file 2: Figure S2. Flow Diagram of Study Participants. (DOCX 50 kb)
Additional file 3: Table S1. Quality of life scores of stroke survivors assessed by Stroke Specific Quality of Life Scale (SSQOLS). (DOCX 15 kb)
Additional file 4: Table S2. Baseline characteristics of informants of FGDs and In-Depth interviews. (DOCX 15 kb)

## Abbreviations

AKUH: Aga Khan University Hospital
CABG: Coronary artery by pass grafting
CT scan: Computerized tomography
CVI: Content validity index
DUHS: DOW University Hospital
FAM: Functional assessment measures
FGD`S: Focus group discussion
GBD: Global burden of disease
HRQOL: Health related quality of life
IDI: In-depth interviews
IQR: Inter-quartile range
LMIC: Low to middle income countries
MRI: Magnetic resonance imaging
mRS: Modified rankin scale
NCDs: Non- communicable disorders
NIHSS: National institute of health stroke scale
QOL: Quality of life
S.D: Standard deviation
SSQOLS: Stroke specific quality of life scale
WHO: World Health Organization
