# Supplementary material for: Quality of life after stroke in Pakistan
Source: BMC Neurol. 2016 Dec 3;16:250. doi: 10.1186/s12883-016-0774-1 (PMC5135839; doi:10.1186/s12883-016-0774-1)
Supplement: Additional file 1: Figure S1. — Conceptual Framework (Adopted from the WHO [World Health Organization]). (DOCX 33 kb) [file 12883_2016_774_MOESM1_ESM.docx]

**Quality Of Life after Stroke in Pakistan: Online Additional Supplementary Files**

Additional File No 1:

Figure 1: Conceptual Framework(Adopted From the WHO[World Health Organization])

**Quality of life of stroke survivors assessed by Stroke Specific Quality of Life Scale**

- **Socio-demographic factors of stroke patients**

1. Age. 2. Gender. 3. Socio economic status.

4. Education of stroke patients.

- **Environmental & patient safety factors**

1. Fall safety. 2. Washroom usage & safety.

3. Assistance in daily living.

4**.** Self-care.

- **Social support factors**

1. Family status. 2. Marital status. 3. Social support assessed by Enriched Social support instrument.

- **Access to health care factors**

1. Medical coverage from any organization. 2. Presence of health care professional attendant.

3. Received Alternative medicine treatment. 4. Received Rehabilitation service on regular basis.

**Stroke patient characteristics**

1. Stroke Sub-Types.
2. Duration of stroke in months.
3. Co-Morbid conditions of the patients.
4. Nicotine Addiction.
5. Central obesity.

- **Functional Disability factors of patients**

1. Modified Barthel Index for disability. 2. Functional assessment measure (FAM). 3. National institute of health stroke severity (NIHSS) scale. 4. Modified Rankin score for functional status.

- **Psychosocial factors**

1. Beck Depression Inventory.

2. Recent life change questionnaire for assessment of stressful events.

3. Dementia.

- **Post-stroke complication factors**

**Primary care giver**

- **Demographic characteristics**

1. Age.

2**.** Gender.

3. Relationship to stroke patients.

4. Employment status.

5. Education of primary care givers.

- **Stress and Quality of life of primary care giver factors**

1. Perceived stress scale for assessment of stress

2. RAND-36 assessment of health related quality of life of care givers.

- **Work related Burden of primary care giver**

1. Left job because of providing care to the stroke patients.

2**.** Change in working hours of primary care giver because of providing care to stroke patients.
