# Supplementary material for: Quality of life after stroke in Pakistan
Source: BMC Neurol. 2016 Dec 3;16:250. doi: 10.1186/s12883-016-0774-1 (PMC5135839; doi:10.1186/s12883-016-0774-1)
Supplement: Additional file 2: Figure S2. — Flow Diagram of Study Participants. (DOCX 50 kb) [file 12883_2016_774_MOESM2_ESM.docx]

Additional File No 2:

Figure 2: Flow Diagram of Study Participants.

| 471 Dyads were approached at two study sites  245 Dyads from AKUH  226 Dyads from DUHS  21 Refusals from AKU  25 Refusals from DUHS  201 Dyads were evaluated for eligibility  224 Dyads were evaluated for eligibility  **49 Excluded**  19 Aphasics  11 TIA  9 Demented.  4 Not confirmed diagnosis of stroke.  2 Cancer patients  2 Disable before stroke.  1 With no primary care giver.  1 On Dialysis  **26 Excluded**  7 Aphasics  7 Not confirmed diagnosis of stroke.  3 Less than 18 yrs. of age.  3 With no primary care giver.  3 Demented.  2 On Dialysis.  1 Comatose patient  350 Dyads out of which 175 Dyads were recruited from each study site and were analyzed |
| --- |
