# Supplementary material for: Quality of life after stroke in Pakistan
Source: BMC Neurol. 2016 Dec 3;16:250. doi: 10.1186/s12883-016-0774-1 (PMC5135839; doi:10.1186/s12883-016-0774-1)
Supplement: Additional file 3: Table S1. — Quality of life scores of stroke survivors assessed by Stroke Specific Quality of Life Scale (SSQOLS). (DOCX 15 kb) [file 12883_2016_774_MOESM3_ESM.docx]

**Additional File no 3:**

**Table 1: Quality of life scores of stroke survivors assessed by Stroke Specific Quality of Life Scale (SSQOLS)**

| *Quality of Life Score of Stroke Patients Assessed by Stroke Specific Quality of life Scale (SSQOLS)* | Mean± S.D |
| --- | --- |
| Mean QOL scores | 164.18±32.30 |
| *Mean QOL scores specified to each domains of SSQOLS* |  |
| Energy | 8.16± 3.21 |
| Family Roles | 9.93± 3.03 |
| Language | 19.21± 6.00 |
| Mobility | 17.24± 7.06 |
| Mood | 18.51± 4.15 |
| Personality | 9.65± 3.06 |
| Social Roles | 12± 4.83 |
| Thinking | 11.96± 2.46 |
| Vision | 12.85± 2.23 |
| Upper-Extremity | 17.87± 5.39 |
| Self-Care Function | 18.302± 5.58 |
| Work Productivity | 8.46± 3.15 |
