# Supplementary material for: Quality of life after stroke in Pakistan
Source: BMC Neurol. 2016 Dec 3;16:250. doi: 10.1186/s12883-016-0774-1 (PMC5135839; doi:10.1186/s12883-016-0774-1)
Supplement: Additional file 4: Table S2. — Baseline characteristics of informants of FGDs and In-Depth interviews. (DOCX 15 kb) [file 12883_2016_774_MOESM4_ESM.docx]

**Additional File no 4:**

**Table 2: Baseline characteristics of informants of FGDs and In-depth interviews**

|  | Mean age in years  (min-max) | Gender | | Attended any teaching program related to stroke |
| --- | --- | --- | --- | --- |
| FGD 1 | 45 (30-63) | Male  Female | 2  2 | 0 |
| FGD 2 | 26.75 (20-40) | Male  Female | 1  3 | 0 |
| FGD 3 | 37.2 (29-48) | Male  Female | 1  4 | 0 |
| In-depth interviews | 43.14 (18-67) | Male  Female | 2  5 | 0 |
